# Parenting stress in parents with and without a mental illness and its relationship to psychopathology in children: a multimodal examination

**DOI:** 10.3389/fpsyt.2024.1353088

**Published:** 2024-02-05

**Authors:** Vanessa Seipp, Klara Hagelweide, Rudolf Stark, Sarah Weigelt, Hanna Christiansen, Meinhard Kieser, Kathleen Otto, Corinna Reck, Ricarda Steinmayr, Linda Wirthwein, Anna–Lena Zietlow, Christina Schwenck, the COMPARE-Family Research Group, Stracke

**Affiliations:** ^1^ Department of Clinical Child and Adolescent Psychology, Justus Liebig University Giessen, Giessen, Germany; ^2^ Department of Rehabilitation Sciences, Technical University Dortmund, Dortmund, Germany; ^3^ Department of Psychotherapy and Systems Neuroscience, Justus-Liebig University Giessen, Giessen, Germany; ^4^ Department of Psychology, Clinical Child and Adolescent Psychology, Philipps-University Marburg, Marburg, Germany; ^5^ Institute of Medical Biometry, University of Heidelberg, Heidelberg, Germany; ^6^ Department of Work and Organizational Psychology, Philipps-University Marburg, Marburg, Germany; ^7^ Department of Psychology, Ludwig-Maximilians-Universität München, Munich, Germany; ^8^ Department of Psychology, Technical University Dortmund, Dortmund, Germany; ^9^ Clinical Child and Adolescent Psychology, Department of Psychology, Technische Universität Dresden, Dresden, Germany

**Keywords:** parents with a mental illness, parenting stress, multimodal, relational schema, psychopathology of children, psychophysiological arousal, fundamental frequency, heart rate

## Abstract

**Objective:**

Children of parents with a mental illness are at heightened risk to develop a mental illness themselves due to genetics and environmental factors. Although parenting stress (PS) is known to be associated with increased psychopathology in parents and children, there is no study investigating PS multimodally in a sample of parents with a mental illness. This study aims to compare PS of parents with and without a mental illness and further to examine the relationship between PS and psychopathology of children.

**Methods:**

Participants were parents with a mental illness and parents without a mental illness and their children aged four to sixteen years. We assessed PS multimodally using a questionnaire, parents’ evaluation of children’s behavior (relational schemas) and psychophysiological arousal of parents during free speech task.

**Results:**

Self-reported PS was increased, and evaluation of children’s behavior was more negative and less positive in parents with a mental illness compared to parents without a mental illness. Children’s psychopathology was associated with self-reported PS and relational schemas of parents. Regarding psychophysiological arousal, parents with a mental illness showed reduced reactivity in heart rate from baseline to free speech task in comparison to parents without a mental illness.

**Conclusions:**

Our findings highlight the importance of implementing intervention programs to reduce PS for parents and children. In particular, parents with a mental illness might benefit from specific intervention programs in order to interrupt the transgenerational transmission of mental disorders.

## 1 Introduction

In Germany, approximately three million children live with a parent with a mental illness (1). Children of parents with a mental illness (COPMI) show three to seven times higher rates of subclinical symptoms (2), poorer health-related quality of life (3) and lower academic achievement (4) compared to children of parents without a mental illness (COPWMI). Due to genetics as well as environmental factors, COPMI are particularly at risk of developing a mental disorder themselves (5, 6). Compared to COPWMI, this risk is two to five times higher (7). Therefore, a transgenerational transmission of mental disorders (TTMD) can be assumed. The association between the parental mental illness and children’s psychopathology depends on different variables and risk constellations (8). Thus, COPMI constitute a high-risk group that needs to be identified and addressed by prevention programs in order to reduce their risk and prevent future mental disorders in this group (6). The model of the TTMD identifies five transmission mechanisms and their interaction related to parent, child, family and social environment contributing to the heightened risk of COPMI to develop a mental disorder themselves (9). Besides genetics, prenatal and social factors, parenting is considered to be a core mechanism in TTMD (9, 10). Parenting is comparably easy to address through preventive measures and interventions. However, there is a lack of studies investigating parenting in COPMI (e.g., (11)), and thus it remains open how this should be targeted optimally. In this context, parenting stress (PS) is of particular importance because it is associated with maladaptive parenting (12) and psychopathology of parents and children (13–15). One purpose of this study, therefore, is to examine PS using multimodal data in parents with and without a mental illness.

## 2 Background

### 2.1 Parenting stress

According to Bowlby’s attachment theory (16), attachment determines how children can regulate their emotions and behavior. While secure attachment is associated with positive developmental outcomes in children (17), insecure attachment is associated with negative outcomes in children and is considered a risk factor for developing a mental illness (17, 18). Positive parenting (e.g. sensitive parenting) provides the foundation for a secure parent-child bond and is considered to be the cornerstone of children’s biological, cognitive, social, and emotional development (19, 20). Stress related to parenting is a normal response experienced by all parents at times (21). It can be helpful as it prompts the use of resources to support positive parenting behaviors (22). It is rather the cumulative impact of stress that adversely affects parenting behavior, the parent-child-interaction and children’s development (21, 23–25). PS is defined as “a set of processes that leads to aversive psychological and physiological reactions arising from attempts to adapt to the demands of parenthood” resulting in negative feelings and beliefs regarding the self and the child (25). There are several theories that help explain how PS affects well-being of parents and children. Abidin (22) postulates that high level of PS reinforces the use of maladaptive parenting behavior and thus negatively influences the development of children, which is confirmed by several studies (12, 26–29). Furthermore, the model of Abidin (22) postulates that aspects of parents, children and environment affect PS. Regarding environmental aspects, several studies highlight the link between PS and social support (30), partnership satisfaction (31) and socioeconomic status (30). Regarding aspects of children, research showed that insecure attachment (26) and internalizing as well as externalizing behavior problems (32) are associated with PS. With respect to aspects of parents, several studies found that parental psychopathology plays a significant role (33–35). According to the transactional model (36), stress has an impact on a behavioral level as well as on a cognitive and affective level. In addition to behaviors, cognitions and affects can also be passed on from parents to children (10) suggesting cognitive processes play an important role how PS affects child outcomes. Additionally, psychophysiological theories can help to explain how PS leads to physiological stress responses in parents and adversely affects interacting with their children (37, 38). Another purpose of this study, therefore, is to examine how cognitive and psychophysiological aspects of PS are associated with psychopathology in children.

### 2.2 Relational schemas

High levels of stress are associated with automatic and rigid rather than controlled and flexible information processing (39, 40). Thus, parents with high levels of PS are less able to understand the child’s behavior within the actual context and are more likely to evaluate the child’s behavior in a more negative way (39, 40). In line with this, studies that found that parents with high levels of PS tend to perceive their child as more difficult and subsequently display more negative affectivity towards it (41–43). A growing body of literature confirms the relation between PS and parental negative attributions of child behavior (41, 44–46). Moreover, a study found that negative attributions mediated the association between PS and maladaptive parenting behaviors (41). Relational schemas (RS) guide parent’s attributions of the intent of their child’s behaviors (47). RS are described as an overlearned and unconscious semantic schema guiding actions and reactions to interpersonal events (48). The interpretation that a child is provoking or intentionally frustrating can be called a negative RS (49). In contrast to that, the interpretation of the child’s behavior as compliant or responsive to the caregiver can be called a positive RS (49). Besides, RS include parent’s sets of implicit beliefs and affective attitudes towards their children (50). Parents’ internal representations can be assessed verbally during the Five-Minute Speech Sample (FMSS; (51)). The FMSS provides independent data that are free from biases associated with self-reports (52). Because RS are part of an unconscious, automatic cognitive process and operate largely outside of the caregiver’s awareness (49), Bullock and Dishion (50) developed the Family Affective Attitude Ratings Scale (FAARS) to identify RS expressed by parents during FMSS (51).

### 2.3 Psychophysiological arousal

Besides information processing, high levels of stress also have an influence on physiological arousal. A key system of physiological arousal is the autonomic nervous system (ANS) which subdivides into the sympathic (SNS) and parasympathic nervous system (PNS; (53)). During stress, SNS produces increased physiological arousal, such as increased heart rate (HR), whereas during periods of stability PNS lowers HR. Heart rate variability (HRV) is a measure of the interplay between SNS and PNS on the HR, and a flexible HRV allows for modulation of arousal depending on situational demands (53). Since emotion regulation depends on flexible adjustment of ANS, HRV is known to be an important index (53). Regarding parent-child-interaction, stress can adversely affect the parent’s ability to regulate emotions (37) and to respond appropriately to the child’s needs (38). A “spillover effect” can decrease emotion regulation skills in children (54) and can consequently lead to increased psychopathology (55). There are some studies suggesting a relation between parenting behaviors and physiological reactivity in parents (38, 56–60). However, the direction of this relation is contradictory. On the one hand, there is evidence that increased reactivity in parents is associated with maladaptive parenting behavior (58, 59). On the other hand, studies found decreased reactivity in parents associated with maladaptive parenting behavior (38, 56, 57, 60). Reasons for the inconsistent results may be found in differences in laboratory tasks, physiological indicators or analytic strategies (60). Fundamental frequency corresponds to the perceived voice pitch and it is determined by the frequency with which the vocal folds open and close when forming sounds (61, 62). Fundamental frequency correlates strongly with established indicators of ANS (e.g. heart rate, blood pressure, and cortisol) and with self-reported emotional state (63, 64). A study confirmed that higher fundamental frequency range was associated with higher emotional arousal (64). While there are some studies examining fundamental frequency in couple relationships (61, 65, 66), there are only a few studies dealing with parent-child relationships or mental illness. A study showed that during conflict talks between adolescents and their parents, higher range of fundamental frequency was associated with higher cortisol levels and more self-reported negative emotionality of parents and adolescents (63).

### 2.4 Parenting stress and psychopathology of parents and children

According to the model of Abidin (22), PS is linked to the psychopathology of both parents and children. Psychopathology increases vulnerability to stress because it reduces access to coping skills that are necessary to decrease stress levels (67). Therefore, a mental illness diminishes the parent’s resources (68). Moreover, the use of ineffective coping strategies can lead to chronically high levels of PS in parents with a mental illness (22, 69). Several studies confirmed the relationship between PS and depression and anxiety symptoms (33–35, 70–74). Independently from psychopathology in parents, a growing body of literature found PS to be related to several negative child outcomes such as internalizing and externalizing behavior problems (75, 76). Longitudinal studies suggest that the relationship between children’s psychopathology and PS is bidirectional (13, 14). Thus, elevations in children’s behavior problems lead to increased PS, which then leads to increased behavior problems in children. Additionally, a longitudinal study found that children’s externalizing behaviors decreased if PS does and vice versa (77). Fredriksen et al. (70) found a mediating effect of PS between parental depressive symptoms and negative child outcomes in a longitudinal study. The findings suggest that PS plays an important role in TTMD. Noteworthy, the research mentioned above used self-report measures only to assess parental and child psychopathology as well as PS that can lead to biased information and compromises objectivity (11). Parents with more psychopathological symptoms might have stronger negative self-appraisals, which lead to elevated reports of PS and psychopathology of children (78). Furthermore, most studies investigated parents with sub-clinical psychopathological symptoms (33). Future research needs to investigate PS multimodally in parents with clinically relevant diagnosis (33, 69, 78). This is especially true regarding the role of PS in TTMD. Therefore, research on parental information processing, as interpretation and beliefs about their children’s behavior is of particular interest. In this term, assessing RS of parents during FMSS can provide an independent data source that is free from biases associated with self-reports (49). Recent studies found that less positive and more negative RS of parents are associated with higher psychopathology in children (49, 79, 80). Moreover, a study found an association between negative RS and increased stress and psychopathology in parents in a clinical-referred child sample (79). Besides, psychophysiological arousal of parents can provide an objective measure of PS (69, 81, 82). To the best of our knowledge, there is no study investigating RS and psychophysiological arousal of parents with a mental illness. Comparing parents with and without a mental illness allows examining psychopathology as a specific risk factor for PS. Since PS is considered a relevant factor in TTMD, it is of clinical importance to gain detailed insight in this relation. This is especially true because PS displays a target for intervention. Examining how PS relates to psychopathology in children regardless of a parental mental illness allows developing specific intervention programs to reduce the risk for negative child developmental outcomes.

### 2.5 The current study

The current study aims to examine parenting stress in parents with a mental illness compared to parents without a mental illness. In order to gain detailed insight into how cognitive and psychophysiological aspects of parenting stress adversely correlates to child outcomes, we investigate the relation of parenting stress and psychopathology in children regardless of a parental mental illness. We examine parenting stress multimodally by self-report, parents’ relational schemas and psychophysiological data.

We aim to address the following hypotheses:

1. Parents with a mental illness report higher parenting stress than parents without a mental illness.
2. Increased self-reported parenting stress is positively correlated with psychopathology in children regardless of parents’ diagnostic status.
3. Parents with a mental illness show more negative and less positive relational schemas than parents without a mental illness.
4. Valence of relational schemas are negatively correlated with psychopathology in children regardless of parents’ diagnostic status.
5. Parents with a mental illness show reduced reactivity in heart rate variability and heart rate from baseline assessment to Five Minute Speech Sample compared to parents without a mental illness.
6. Parents with a mental illness show higher fundamental frequency range during the Five Minute Speech Sample than parents without a mental illness.
7. Psychophysiological arousal in parents is correlated with psychopathology in children regardless of parents’ diagnostic status.

## 3 Method

### 3.1 Participant recruitment and study inclusion and exclusion criteria

The current study is part of the German prospective multicentre RCT *Children of Mentally Ill Parents At Risk Evaluation* (COMPARE-family; grant number: 01GL1748B) and its add on-project COMPARE-emotion (grant number: 01GL1748C and 01GL1748E). For detailed description of this study, see study protocols (5, 83). Parents with a mental illness were recruited as part of the prospective multicenter RCT COMPARE-family (5, 83). Patients were recruited from the outpatient clinics of the universities of Gießen, Bochum, Marburg and Landau. In the study centers, we used the universities’ mailing lists, mailings of families with children in the corresponding age range provided by local registry offices, and public advertisement (flyer, newspaper, online-platforms) as recruitment tools. Parents without a mental illness were recruited as part of the add-on project COMPARE-emotion in study centers Gießen and Dortmund. Parents without a mental illness and their children were recruited by mailings of children in the corresponding age range provided by local registry offices, public advertisement (flyer, online-platforms), and the research group’s database of former study participants. Inclusion criteria for COMPARE-emotion were: a) children between 4 to 16 years of age; b) parents agreeing to participate in a videotaped paradigm; c) parents seeking psychotherapeutic treatment and meeting diagnostic criteria of a mental illness according to the Diagnostic and Statistical Manual of Mental Disorders (DSM-5; (84)) for parents with a mental illness; or d) parents with no mental illness and no psychotherapeutic treatment during the life of parents without a mental illness; e) children living with the participating parent for at least half of the week.

Exclusion criteria were a) insufficient German language skills; b) children presenting severe impairment requiring urgent treatment; c) parental ongoing outpatient or inpatient treatment; d) regular use of benzodiazepines as is thought to hamper cognitive behavioral therapy; e) parents without a mental illness reporting psychopathological symptoms above the cut-off value in the Brief Symptom Inventory (85); f) COPWMI meeting diagnostic criteria according to the DSM-5 (84). Local ethics committees at all participating universities approved the study. All participants provided written informed consent. For participation, each child received a gift or a financial allowance of €5. Parents without a mental illness and their children participated once, while parents with a mental illness and their children participated in repeated assessments at three measurement points (83). For the current study, we analyzed data of the first assessment point in parents with a mental illness participating in the study centers Giessen, Bochum, Marburg and Landau.

### 3.2 Procedure

Before participating in the laboratory assessment of COMPARE-emotion, families provided written informed consent and completed several questionnaires online. Assessments took place at the laboratories of the Universities of Gießen, Dortmund, Marburg and Landau between 2018 and 2022 and lasted one hour. During the assessment, we conducted different paradigms with the dyads including the FMSS (51) for assessment of RS and fundamental frequency to measure psychophysiological arousal in parents. Due to availability of the ECG-equipment, we assessed psychophysiological arousal via HR and HRV exclusively in Gießen. Parents placed three electrodes on themselves (one over the right collarbone and one over each of the lower ribs) at the start of the assessment. Once the ECG signal was registered, parents were asked to sit quietly and describe a picture puzzle for one minute. After completion of the baseline period, parents performed the FMSS. Here, parents were asked to speak for five minutes about their thoughts and feelings regarding their child and how they get along together. We recorded baseline period and speech samples on video camera and converted them to wav-audio files for further analyses.

### 3.3 Participants

We conducted analyses with different sample sizes because not all families participating in laboratory assessment completed the questionnaires of parenting stress and psychopathological symptoms of children. Thus, we included all available data sets and did not exclude families of our analyses because of missing values. Besides, we assessed electrocardiographic (ECG) activity exclusively in the study center Gießen. Demographic information for sub-samples are described below.

Five Minute Speech Samples of 189 independent parent-child dyads were available (*n* = 91 parents with a mental illness, *n* = 98 parents without a mental illness). Data of the dyads were used for analyses of RS and fundamental frequency. Since not all parents returned the questionnaire, information about psychopathology of children (*n* = 20 in COPMI, *n* = 3 in COPWMI) was missing. Due to technical problems that led to the video-files not being readable, baseline-recordings of parents (*n* = 8) were missing. Of the parents performing the Five Minute Speech Sample, 150 were mothers (79%) and 100 children were female (53%). Children ranged from four to 16 years of age (*M* = 9.08, *SD* = 3.40). Groups did not differ in age, gender or parents’ gender but in parents’ age, parents’ and children’s psychopathological symptoms and socioeconomic status (SES; see **Table 1**). COPMI showed higher psychopathological symptoms than COPWMI. Parents with a mental illness were younger and their SES was lower. According to Lampert et al. (86), the SES of parents without a mental illness can be classified as high, while the SES of parents with a mental illness can be classified in the middle status group. We used a structured diagnostic interview to determine mental disorders and verify diagnostic criteria for study inclusion of parents with a mental illness. The most common disorders among parents were Depressive Disorders as primary diagnosis (42%), followed by Trauma- and Stressor-Related Disorders (28%), Anxiety Disorders (23%), Somatic Symptom and Related Disorders (3%), Feeding and Eating Disorders (1%), Personality Disorders (1%), Disturbance of Activity and Attention Disorders (1%) and Schizophrenia Spectrum and Other Psychotic Disorders (1%). The mean number of comorbid diagnosis was two (*SD* = 1.22, range from 1 - 5), and severity of the primary diagnosis ranged from four to eight (*M* = 5.88, *SD* = 0.91) on a 9-point-Likert-Scale. Digits in this range indicate a clinical diagnosis.

**Table 1:** Demographic characteristics of participants and means and standard deviations of psychopathological symptoms of children and parents for analyses with the Five Minute Speech Sample.

	PMI (*N* = 91)	PWMI (*N* = 98)	*t(187)* *χ^2^(1)*	*p*	*η_p_^2^*
	*M* (SD)	*M* (SD)			
Children					
Age	8.94 (3.27)	9.20 (3.52)	-.50	.616	.001
number of girls (%)	44 (48.35)	56 (57.14)	1.46	.226	.008
CBCLext (*T*-score)	52.13 (10.19)^a^	46.82 (8.67)^b^	3.62	<.001	.074
CBCLint (*T*-score)	56.49 (9.32)^a^	48.25 (9.97)^b^	5.42	<.001	.152
Parents					
Age	39.93 (6.42)	42.65 (6.39)	-2.89	.004	.043
Gender (female, %)	76 (83.52)	74 (76.29)	1.52	.217	.008
SES	14.28 (3.71)	18.27 (2.22)	-9.13	<.001	.306
BSI GSI (*T*-score)	63.37 (16.37)	40.08 (9.07)	15	<.001	.448

CBCLext, Child Behavior Checklist Externalizing Scale; CBCLint, Child Behavior Checklist Internalizing Scale; ^a^n = 71; ^b^n = 95; SES, socioeconomic status; BSI, Brief Symptom Inventory; GSI, Global Severity Index; PMI, parents with a mental illness; PWMI, parents without a mental illness.

Self-reported PS was available from *n* = 54 data sets of parents with a mental illness and *n* = 96 data sets of parents without a mental illness. Of this sub-sample, 118 were mothers (79%) and 79 children were female (53%). Children ranged from four to 16 years of age (*M* = 8.67, *SD* = 3.23). Groups did not differed in gender of children and parents, but in age of children and parents, psychopathological symptoms of children and parents, and SES (see **Table 2**). In this sub-sample, COPMI were younger than COPWMI. The most common disorders among parents of this sub-sample were Depressive Disorders as primary diagnosis (39%), followed by Trauma- and Stressor-Related Disorders (31%), Anxiety Disorders (22%), Somatic Symptom and Related Disorders (4%), Feeding and Eating Disorders (2%) and Schizophrenia Spectrum and Other Psychotic Disorders (2%). The mean number of comorbid diagnosis was two (*SD* = 1.26, range from 1 - 5), and severity of the primary diagnosis ranged from four to eight (*M* = 5.80, *SD* = 0.98).

**Table 2:** Demographic characteristics of participants and means and standard deviations of psychopathological symptoms of children and parents for analyses of self-reported parenting stress.

	PMI (*N* = 54)	PWMI (*N* = 96)	*t(148)/χ^2^(1)*	*p*	*η_p_^2^/φ*
	*M* (SD)	*M* (SD)			
Children					
Age	7.69 (2.31)	9.22 (3.53)	-3.20	<.001	.053
number of girls (%)	24 (44.4)	55 (57.3)	2.29	.130	-.124
CBCLext (*T*-score)	51.19 (10.28)	46.83 (8.62)	2.76	.006	.049
CBCLint (*T*-score)	55.33 (9.43)	48.25 (9.91)	4.27	<.001	.110
Parents					
Age	39.72 (4.92)	42.68 (6.41)	-3.15	.002	.140
number of mothers (%)	44 (81.5)	74 (77.1)	0.40	.528	.052
SES	15.14 (3.11)	18.24 (2.22)	-6.46	<.001	.360
BSI GSI (*T*-score)	62.33 (13.33)	40.12 (9.09)	10.90	<.001	.448

CBCLext, Child Behavior Checklist Externalizing Scale; CBCLint, Child Behavior Checklist Internalizing Scale; SES, socioeconomic status; BSI, Brief Symptom Inventory; GSI, Global Severity Index; PMI, parents with a mental illness; PWMI, parents without a mental illness.

ECG recordings were available from *n* = 30 data sets of parents with a mental illness and *n* = 33 data sets of parents without a mental illness. Data of this sample were used for analyses of HR and HRV. Of this sub-sample, 48 were mothers (76%) and 36 children were female (57%). Parents ranged from 24 to 56 years of age (*M* = 41.72, *SD* = 7). Groups did not differed in children’s age, children’s gender or parent’s gender but in age and psychopathological symptoms of parents and SES (see **Table 3**). In this sub-sample, COPMI did not differed in externalizing psychopathological symptoms. The most common disorders among parents of this sub-sample were Depressive Disorders as primary diagnosis (37%), followed by Trauma- and Stressor-Related Disorders (27%) and Anxiety Disorders (27%), Somatic Symptom and Related Disorders (3%), Personality Disorders (3%) and Schizophrenia Spectrum and Other Psychotic Disorders (3%). The mean number of comorbid diagnosis was two (*SD* = 1.97, range from 1 - 5), and severity of the primary diagnosis ranged from four to eight (*M* = 5.93, *SD* = 0.79).

**Table 3:** Demographic characteristics of participants and means and standard deviations of psychopathological symptoms of children and parents for analyses of electrocardiographic activity.

	PMI (*N* = 30)	PWMI (*N* = 33)	*t(61)/χ^2^(1)*	*p*	*η_p_^2^/φ*
	*M* (SD)	*M* (SD)			
Children					
Age	8.79 (3.36)	8.52 (3.27)	.33	.743	.002
number of girls (%)	16 (44.4)	20 (55.6)	.34	.560	-.073
CBCLext (*T*-score)	51.11 (7.65)	48.47 (8.16)	1.28	.207	.028
CBCLint (*T*-score)	56.15 (6.54)	49.16 (7.45)	3.80	<.001	.202
Parents					
Age	39.86 (6.64)	43.41 (6.99)	-2.02	.047	.065
number of mothers (%)	22 (45.8)	26 (54.2)	0.56	.456	-.095
SES	15.51 (2.76)	17.92 (2.71)	-3.50	<.001	.167
BSI GSI (*T*-score)	62 (12.68)	38.67 (9.26)	8.08	<.001	.538

CBCLext, Child Behavior Checklist Externalizing Scale; CBCLint, Child Behavior Checklist Internalizing Scale; SES, socioeconomic status; BSI, Brief Symptom Inventory; GSI, Global Severity Index; PMI, parents with a mental illness; PWMI, parents without a mental illness.

### 3.4 Measures

#### 3.4.1 Parental relational schemas

We assessed parental RS using the Family Affective Attitude Rating Scale (FAARS; (87)). The FAARS is an extension and re-formulation of the original FMSS coding system (51) for parents of children in childhood and adolescence (50). The measures of RS consist of items reflecting negative and positive attitudes relevant for parent-child-relationship. Respective items score on a 9-Point-Likert-Scale from 1 *(not present)* to 5 *(one concrete example)* to 9 *(multiple examples)*. Following the methodology of Bullock and Dishion (50), we averaged respective items reflecting positive RS and negative RS. The scale for positive RS derived from the mean of the five items: parent is positive regarding the child’s behavior, parent is positive regarding the child’s traits, parent reports a positive relationship with the child, parent attributes positive intentions of the child, parent makes statements of caring and loving. For computing the scale of negative RS, we used the mean of the five items: parent is critical of the child’s behavior, parent is critical of the child’s traits, parent reports a negative relationship with the child, parent attributes negative intentions of the child, parent reports of conflict or anger. To assess inter-rater reliability, two raters coded speech samples independently and blind to parental diagnostic status. As recommended by Bullock et al. (87), for our study we calculated inter-rater reliability ratings from 25% of analyzed samples. Indicating good consensus among the coders, intraclass correlations ranged from 0.86 to 0.91 (*M* = 0.89) for negative RS and from 0.95 to 0.97 (*M* = 0.96) for positive RS. Cronbach’s alpha values indicate good internal consistency with negative RS (α = 0.83) and positive RS (α = 0.80). Our results are similar to those reported in previous FAARS validation studies (50, 79, 80).

#### 3.4.2 Fundamental frequency

We assessed parents’ fundamental frequency during a baseline period and FMSS. Prior to calculating fundamental frequency, baseline and FMSS wav-audio files were cleaned for artefacts (experimenter speaking, laughter, etc.) using a cutting program (Audacity, Version 3.0.0). We included the normal range of speech in our analyses by setting the floor at 75 Hz and the ceiling at 300 Hz (88). Using the voice analysis program Praat (Version, 6.1.56; (89)), we assessed minimum and maximum values in 0.25 second intervals. Fundamental frequency range was calculated by subtracting minimum from maximum values for each data set.

#### 3.4.3 Heart rate and heart rate variability

We used the BIOPAC system MP160 (90) and the portable bionomadix modules for assessing electrocardiographic (ECG) activity with an acquisition sample rate of 1000 Hz. 3-point ECG using Ag/AgCl electrodes (one over the right collarbone and one over each of the lower ribs) were installed on parents’ thoraxes. The physiological data were processed using the Acqknowledge software (Version 5.0.8; (90)). We inspected the ECG signals using Kubios HRV Scientific software (Version 4.0.1). The software identified successive R spikes via an automatic beat detection algorithm, we visually inspected and corrected for artefacts. Mean HR was calculated in beats per minute (bpm) based on the ECG channel. We used the time-domain index “root mean square of successive differences between normal heartbeats” (RMSSD) for calculating HRV. We choose RMSSD, because it is less affected by respiratory rate (91). For detailed description of RMSSD see Shaffer and Ginsberg (92).

#### 3.4.4 Parental-stress questionnaire

To assess parental stress experience, we used the German “Parenting-Stress-Questionnaire” versions for children in kindergarten, preschool and school (ESF; (93)). The ESF consists of 38 items rated on a 4-point Likert Scale from 0 *(disagree)* to 3 *(fully agree)*. Items are aggregated into the four scales, parenting stress *(e.g. perceived parenting skills, stress in interaction with the child)*, role restriction *(perceived limitations associated with raising the child)*, social support *(perceived support from relatives, friends and close environment)* and partnership *(e.g. perceived support from the partner, agreement on parenting issues)*. For partnership, the respective items were answered only if the parent was currently living with a partner. Domsch and Lohaus (93) reported internal consistencies ranging from acceptable (Cronbach’s alpha = 0.76 for social support) to very good (Cronbach’s alpha = 0.92 for parental stress).

#### 3.4.5 Psychopathology of children

We used the German versions of the parent-reported questionnaire *Child Behaviour Checklist (CBCL).* Depending upon the children’s age to assess psychopathology of COPMI and COPWMI, we applied the CBCL 1½-5 (94) or the CBCL 6-18R (95). The German version of the CBCL 1½-5 (94) assesses problems of children between 1,5 and 5 years of age. It includes 99 items rated on a 3-point Likert Scale from 0 *(never or not true)* to 2 *(often or very true)*. The German version of CBCL 6-18R captures problems of children and adolescents ageing from 6 to 18 years. It includes 120 items rated on a 3-point Likert Scale from 0 *(never or not true)* to 2 *(often or very true)*. In both versions, items are aggregated into three superordinate scales (externalizing problems, internalizing problems and total problems). Achenbach and Rescorla (94) reported good to very good internal consistencies for CBCL 1½-5 (Cronbach’s alpha = 0.89 - 0.95) as well as Döpfner et al. (95) for CBCL 6-18R (Cronbach’s alpha = 0.82 - 0.93).

#### 3.4.6 Diagnostic status of parents and children

We used the German version of the self-reported questionnaire *Brief Symptom Inventory* (*BSI* (85);) to assess psychopathology of parents with and without a mental illness. The BSI contains 53 items rated on a 5-point Likert Scale from 0 *(not at all)* to 4 *(very much)*. We aggregated items into Global Severity Index (GSI). Derogatis (85) reported internal consistency of GSI to be very good (Cronbach’s Alpha = .97). We invited parents without a mental illness to further diagnostic examination and excluded them of our analyses if the GSI was above the cut-off value (*T*_GSI_ ≥ 62). The *Diagnostic Interview for Mental Disorders* (*DIPS*; (96)) is a structured diagnostic interview to determine mental disorders according to the DSM-5 (84). We used the DIPS to verify diagnostic criteria for study inclusion of parents with a mental illness. The interview has a good to very good inter-rater reliability (97). The *Diagnostic Interview for Mental Disorders During Childhood and Adolescence* (*Kinder-DIPS;* (98)) and the *Structured Interview for Preschool Age (SIVA*; (99)) are structured diagnostic interviews to determine mental disorders from age of six to adulthood (Kinder-DIPS) as well as in preschool children (SIVA) according to DSM-5 (84). We used the parent-report versions of both for children’s diagnostic assessment. Good to very good interrater reliabilities are reported for both the Kinder-DIPS (100) and SIVA (99). In COPMI, we conducted diagnostic interviews by default. In COPWMI, we conducted diagnostic interviews if total problem scale of CBCL was above the cut-off value (*T*_CBCLSum_ ≥ 60).

#### 3.4.7 Socioeconomic status

We assessed *SES* of parents with and without a mental illness according to the KiGGS study (101). The SES-index was calculated as a point-sum score from 1 to 7 based on parents’ information on their schooling and occupational qualifications (*1 = not finished school and professional training to 7 = high school SAT-level and university degree*), occupational status *(1 = in professional training or unskilled employee to 7 = executive or self-employed)*, and net equivalent income *(1 = less than €1250 net income per month to 7 = more than €5000 net income per month)*. This allows to calculate a multidimensional SES-index ranging between 3.0 and 21.0 (86). Alternatively, SES can be categorized into status groups from low (range: 3.2 -8.7) to high (range: 17.0 – 21.0), each including 20% of children and adolescents of a representative German sample. The more broadly defined middle status group (range: 8.8 – 16.9) includes 60% of children and adolescents of a representative sample (86). We used this categorization to compare the SES of our study’s samples with a representative sample. Based on the mean SES of our samples, parents with a mental illness and their children can be assigned to the middle status group, whereas the mean SES of parents without a mental illness and their children can be categorized as high (see **Tables 1**–**3**).

### 3.5 Statistical analyses

We performed statistical analyses using SPSS version 28.0 (102). To address the need for confounding variables in further analyses, we examined possible differences in demographic characteristics between groups (two-sample *t*-test or chi-square test, respectively; see **Tables 1**–**3**). Since groups differed on SES, parents’ and child age, we conducted correlation analyses with all dependent variables. Since studies have shown that PS is associated with SES (30) and because of significant correlations with self-reported PS (*p* <.05), we included SES as a covariate in respective analyses. Furthermore, we found significant correlations of child age and self-reported PS (*p* <.05). Since studies produced mixed findings regarding the association of age of children and PS (30, 77), we decided to include child age as a covariate in respective analyses to minimize potential biases in our results.

We applied an alpha level of 0.05 (two-tailed) and calculated effect sizes (*η_p_* ^2^) for all statistical analyses. As multiple testing leads to an inflation of the alpha error and hence an increased likelihood of type 1 errors, we used Bonferroni-Holm correction for hypotheses 1 to 4, 6 and 7. The described *p*-values for the inferential statistical tests are already adjusted and can be compared with the applied alpha level. Due to missing values in questionnaires, we examined if parents reporting higher psychopathological symptoms during the DIPS (96) were more likely not to return the questionnaires. For that, we conducted a *t*-test for independent samples in corresponding analyses. In addition, we performed analyses of questionnaire data with multiple imputations in order to test the robustness of our results. In general, results did not differ between analyses with and without multiple imputations.

To test for differences in self-reported PS between groups (Hypothesis 1), we conducted a multivariate analysis of covariance (MANCOVA) with group (parents with vs. without a mental illness) as a between-subject variable. SES and age of children were included as covariates. Scales of self-reported PS were dependent variables. We used Pillai’s trace as the statistic measure to test for significance. Significant effects were decomposed using univariate ANCOVAs.

To examine the association of self-reported PS and children’s psychopathology (Hypothesis 2), we applied a partial correlation analysis with group, SES and age of children as control variables. We used Pearson’s *r* correlation coefficients as statistic measures.

To test for differences in parental RS in both groups (Hypothesis 3), we conducted a multivariate analysis of variance (MANOVA) with group (parents with vs. without a mental illness) as a between-subject variable and parents’ positive and negative RS as dependent variables. We used Pillai’s trace as the statistic measure to test for significance. Significant effects were decomposed using univariate ANOVAs.

To examine the association of parental RS and children’s psychopathology (Hypothesis 4), we conducted a partial correlation analysis with group as a control variable. We used Pearson’s *r* correlation coefficients as statistic measures.

To test for differences in fundamental frequency range in both groups (Hypothesis 5), we applied an ANOVA with repeated measures with group (parents with vs. without a mental illness) as between-subject factor and fundamental frequency range of phase (baseline, FMSS) as dependent variable, which we entered as a within-subject factor. We used the *F*-statistic as a statistic measure to test for significance.

To test for differences in HR and HRV in both groups (Hypothesis 6), we conducted a MANOVA with repeated measures with group (parents with vs. without a mental illness) as between-subject factor. Mean HR and HRV of each phase (baseline, FMSS) were dependent variables, which we entered as within-subject factors. We used Pillai’s trace as the statistic measure to test for significance. Significant effects were decomposed using univariate ANOVAs.

To examine the association of parent’s psychophysiological arousal and children’s psychopathological symptoms (Hypothesis 7), we applied a partial correlation analysis with group as a control variable. We included parents’ psychophysiological arousal during FMSS (fundamental frequency range, HR and HRV) and children’s psychopathological symptoms in partial correlation analysis. We used Pearson’s *r* correlation coefficients as statistic measures.

## 4 Results

### 4.1 Self-reported parenting stress of parents with and without a mental illness

Regarding self-reported PS, a significant group difference with a large effect size was found in MANCOVA, *V =* 0.19, *F*(4, 143) = 8.33, *p* <.001, *η_p_^2^* = .189. However, univariate ANCOVAs on the outcome variables revealed non-significant group differences on PS, *F*(1, 146) = 0.21, *p* = .644, and on role restriction, *F*(1, 146) = 2.39, *p* = .124. We found a significant group difference on social support, *F*(1, 146) = 28.42, *p* <.001, and support in parental relationship, *F*(1, 146) = 6.10, *p = .*045, with parents with a mental illness reporting less social support and less support in parental relationship (for means and standard deviations, see **Table 4**). 15% of parents with a mental illness (*n* = 8) reported living without a partner, whereas this was reported by 4% of parents without a mental illness (*n* = 4). Due to missing values of self-reported PS in parents with a mental illness (*n* = 54, 41% missing), we examined if parents reporting higher psychopathological symptoms during the diagnostic interview were more likely not to complete the questionnaire. For that, we conducted a *t*-test for independent samples with group (questionnaire complete vs. missing) as independent and severity of primary diagnosis as dependent variable. Results indicated that groups do not differ in severity of psychopathological symptoms, *t*(89) = -1.056, *p = .*294, *η_p_* ^2 =^ 012.

**Table 4:** Mean scores (in Stanine) and standard deviations of self-reported parenting stress.

Scale	PMI (*N* = 54)		PWMI (*N* = 96)	
	*M*	*SD*	*M*	*SD*
Parenting Stress	5.11	2.59	5.63	2.81
Role Restriction	5.02	2.31	5.49	2.55
Social Support	4.50	1.85	6.85	2.32
Partnership	4.11	2.38	6.52	2.68

Socioeconomic status and age of children were included as covariates. PMI, parents with a mental illness; PWMI, parents without a mental illness.

### 4.2 Relation of self-reported parenting stress and psychopathological symptoms of children

We conducted partial correlation analyses of self-reported PS, CBCLint, and CBCLext with group, age of children and SES as control-variables (see **Table 5**). We found significant correlations of CBCLext with self-reported PS. All significant correlation coefficients were in the expected direction with positive associations between psychopathological symptoms and PS. Significant correlation coefficients can be classified as moderate (103).

**Table 5:** Partial correlation analysis of self-reported parenting stress, parent’s relational schema, and children’s psychopathological symptoms.

	Parenting Stress^a^	Role Restriction^a^	Social Support^a^	Partnership^a^	Negative Relational Schema^b^	Positive Relational Schema^b^
CBCLint	.12	.17	-.19	-.10	.19*	-.19*
CBCLext	.38**	.31**	.05	-.03	.30**	-.29**

^a^n = 150, socioeconomic status, age of children and group were included as covariates. ^b^ n = 166, group was included as a covariate. * p <.05 ** p <.01. Tests of significance two-tailed. CBCLint, Child Behavior Checklist Internalizing Scale; CBCLext, Child Behavior Checklist Externalizing Scale.

### 4.3 Relational schemas of parents with and without a mental illness

To test for differences in talking time during FMSS, we conducted a *t*-test for independent samples with group (parents with vs. without a mental illness) as independent and talking time (in minutes) as dependent variable. There was a significant group difference (*t*(187) = -2.59, *p* = .010). Parents with a mental illness talked for a shorter time (*M* = 2.96, *SD* = .90) compared to parents without a mental illness (*M* = 3.31, *SD* = .92). Since parents differed in terms of their talking time during FMSS, we conducted correlation analyses with the dependent variables. Because of non-significant correlations with negative RS (*p* = .357) and positive RS (*p* = .887), we did not include parents’ talking time as a covariate in analyses of RS.

Results of MANOVA indicated that parents with a mental illness differed from parents without a mental illness regarding parents’ RS with a large effect size (*V =* 0.29, *F*(2, 186) = 38.50, *p* <.001; *η_p_^2^* = .293). Univariate ANOVAs revealed significant group differences on negative RS, *F*(1, 187) = 66.47, *p* <.001, and positive RS, *F*(1, 187) = 52.01, *p* <.001, with parents with a mental illness reporting more negative and less positive RS (for means and standard deviations, see **Table 6**).

**Table 6:** Mean scores and standard deviations of parent’s relational schemas.

	PMI (*N* = 91)		PWMI (*N* = 98)	
	*M*	*SD*	*M*	*SD*
Negative Relational Schema	2.45	1.24	1.34	0.50
Positive Relational Schema	3.62	1.24	4.81	1.02

PMI, parents with a mental illness; PWMI, parents without a mental illness.

### 4.4 Relation of parent’s relational schema and children’s psychopathological symptoms

We conducted partial correlation analyses of negative and positive RS, CBCLint and CBCLext with group as control-variable (see **Table 5**). We found significant correlations of CBCLext and CBCLint with parents’ RS. All significant correlation coefficients were in the expected direction with valence of RS associated with higher psychopathological symptoms and can be classified as low (103). Due to missing values of psychopathological symptoms in COPMI (*n* = 20, 22% missing), we examined if parents reporting higher psychopathological symptoms during the diagnostic interview were more likely to not complete the CBCL. For that, we conducted a *t*-test for independent samples with group (CBCL complete vs. missing) as independent and severity of primary diagnosis as dependent variable. Results indicated that groups do not differ in severity of psychopathological symptoms, *t*(89) = -.956, *p = .*342, *η_p_* ^2^ = 010.

### 4.5 Fundamental frequency of parents with and without a mental illness

Results of mixed ANOVA revealed a significant effect of phase, *F*(1, 178) = 24.50, *p* <.001, *η_p_^2^* = .121, indicating that fundamental frequency ranges differed between baseline period and FMSS, with higher fundamental frequency ranges during baseline period. Groups did not differ in fundamental frequency ranges, *F*(1, 178) = 1.66, *p = .*199, *η_p_^2^* = .009, nor did we find an interaction effect between group and phase, *F*(1, 178) = 1.10, *p = .*297, *η_p_^2^* = .006. Since baseline-period was missing in *n* = 8 data sets, this analysis was conducted with a slightly reduced sample sizes. For means and standard deviations, see **Table 7**.

**Table 7:** Mean scores and standard deviations of parent’s fundamental frequency range, HR and HRV per phase.

	PMI		PWMI	
	*M*	*SD*	*M*	*SD*
fundamental frequency range (Hz)^a^				
Baseline	43.10	12.94	46.09	13.62
FMSS	40.75	11.58	42.47	13.06
Mean Heart Rate (bpm)^b^				
Baseline	80.97	9.83	80.48	9.48
FMSS	81.63	9.11	83.15	10.28
Heart Rate Variability (RMSSD)^b^				
Baseline	26.44	14.16	28.06	16.55
FMSS	27.05	13.95	26.74	14.01

PMI, parents with a mental illness; PWMI, parents without a mental illness. ^a^ n = 84 for PMI, n = 96 for PWMI ^b^ n = 30 for PMI, n = 33 for PWMI. bpm, beats per minute; FMSS, Five Minute Speech Sample; Hz, Hertz; RMSSD, root mean square of successive differences between normal heartbeats.

### 4.6 Heart rate and heart rate variability of parents with and without a mental illness

Results of mixed MANOVA indicated a significant effect of phase with a large effect size, *V = .*25, *F*(2, 60) = 9.90, *p* <.001, *η_p_^2^* = .248, but no significant effect of group, *V = .*00, *F*(2, 60) = .089, *p = .*915, *η_p_^2^* = .003. However, we found a significant interaction between group and phase with a moderate effect size, *V = .*10, *F*(2, 60) = 3.44, *p = .*039, *η_p_^2^* = .103.

**Figure 1:**
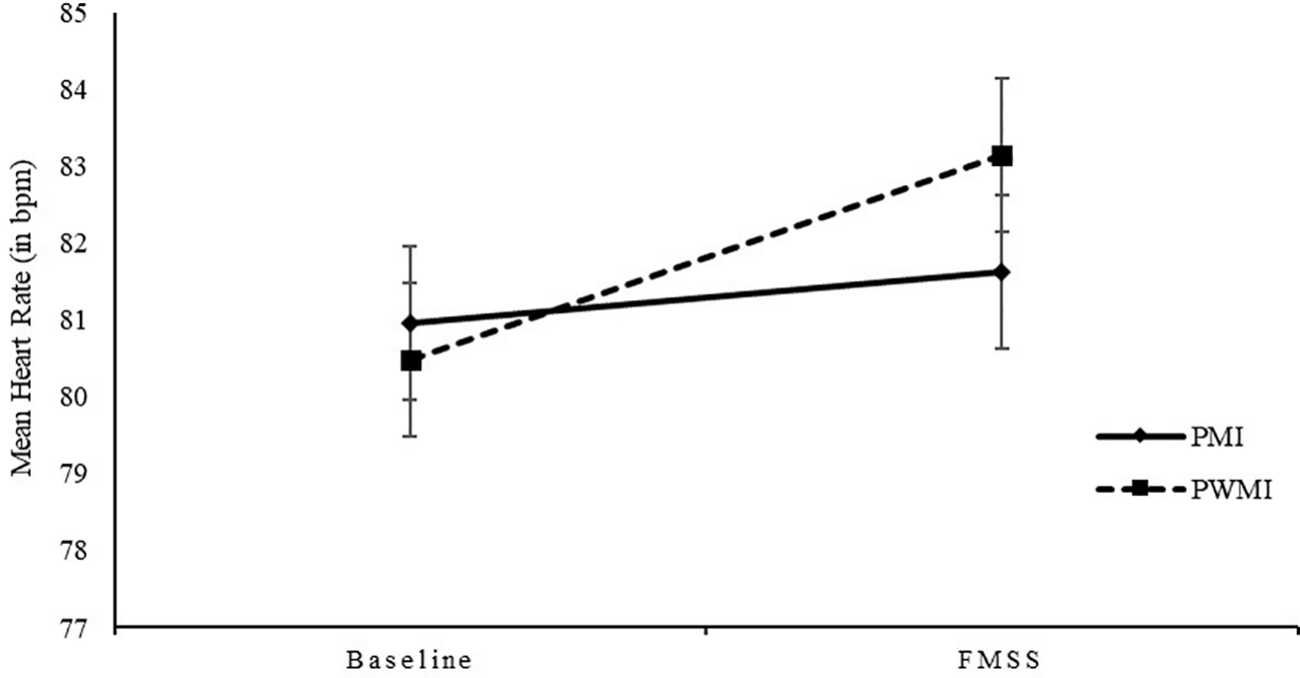
Mean heart rate (bpm) and standard deviations for parents with and without a mental illness at baseline and during FMSS. bpm, beats per minute; PMI, parents with a mental illness; PWMI, parents without a mental illness; FMSS, Five Minute Speech Sample.

Results of univariate ANOVA revealed a significant effect of phase in mean HR, *F*(1, 61) = 18.49, *p* <.001, *η_p_^2^* = .233, indicating that mean HR differed between baseline and FMSS, with a higher mean HR during FMSS. We found a significant interaction between group and phase, *F*(1, 61) = 6.66, *p = .*024, *η_p_^2^* = .098, indicating reduced reactivity in mean HR from baseline to FMSS in parents with a mental illness compared to parents without a mental illness (see **Figure 1**). For means and standard deviations, see **Table 7**.

Results of univariate ANOVA for HRV revealed no significant effect of phase, *F*(1, 61) = 0.34, *p = .*564, *η_p_^2^* = .005, no significant effect of group, *F*(1, 61) = 0.03, *p = .*858, *η_p_^2^* = .001, nor a significant interaction of group and phase, *F*(1, 61) = 2.48, *p = .*121, *η_p_^2^* = .039. For means and standard deviations, see **Table 7**.

### 4.7 Relation of parent’s psychophysiological arousal and children’s psychopathological symptoms

We conducted partial correlation analyses of fundamental frequency range, mean HR and HRV, CBCLint and CBCLext with group as control-variable. We did not find any significant correlations of parent’s psychophysiological arousal and children’s psychopathological symptoms.

## 5 Discussion

In this study, we investigated PS using multimodal data in parents with and without a mental illness. We expected parents with a mental illness to report higher levels of PS than parents without a mental illness. Since PS is considered a mechanism in TTMD, we aimed to examine how PS affects psychopathological symptoms in children. We expected self-reported PS to be positively correlated with children’s psychopathological symptoms regardless of a parental mental illness. Regarding the way parents think about their children, we expected parents with a mental illness to show more negative and less positive RS. We expected negative RS to be positively correlated with children’s psychopathological symptoms and positive RS to be negatively correlated with children’s psychopathological symptoms. Furthermore, we expected parents with a mental illness to show reduced reactivity from the baseline-period to FMSS in mean HR and HRV and higher fundamental frequency ranges during FMSS. Finally, we examined whether psychophysiological arousal in parents is correlated with psychopathological symptoms in children.

### 5.1 Self-reported parenting stress in parents with a mental illness and the relationship to psychopathological symptoms in children

Our study is the first to compare PS using multimodal data in a sample of parents with and without a mental illness and investigating the relationship of PS with psychopathological symptoms of their children. Confirming our hypothesis, we found parents with a mental illness to report higher levels of PS than parents without a mental illness. This is in line with previous studies on parents with depression and anxiety symptoms (30, 33–35, 70–74, 88). Taking a closer look at the questionnaire’s scales, we found parents with a mental illness to perceive less social support and less support in parental relationship. This is in line with findings that suggest that parents with a mental illness report a below-average extent of social support (104). Additionally, over 50% of parents in this study reported a need for help, mostly with childcare. The extent of social support and need for help were moderated by sociodemographic differences (104). That is, in general, mothers report a higher extend of needed help compared to fathers, and unmarried patients report less social support than married ones (104). This could also be the case in our sample as 79% of the participants were female. Moreover, 15% of parents with a mental illness versus 4% of parents without a mental illness reported living without a partner. Besides, social support was found to increase parenting qualities in parents with a mental illness and therefore reducing the risk for TTMD in COPMI (for review see (105). According to Bowlby’s attachment theory (16), positive parenting provides the foundation for a secure attachment, which is linked to positive developmental outcomes in children. Thus, specific programs to increase social support in parents may be useful to support the use of positive parenting and to reduce the heightened risk for TTMD in COPMI. In line with our hypothesis, we found associations between PS and children’s psychopathological symptoms regardless of parents’ diagnostic status. This is in line with theories of PS (22) that PS has an influence on the development of children. Taking a closer look, we found significant associations between children’s externalizing psychopathological symptoms and PS as well as role restriction. Correlation coefficients were in the expected direction with higher psychopathological symptoms associated with higher PS and role restriction. This is in line with previous research finding PS to be related to externalizing problems in children (75, 76). However, we did not find any associations with children’s internalizing symptoms. Since we assessed age of children as a covariate, it could be that the relation between PS and children’s internalizing symptoms is more relevant for a particular age group. It is important to note that longitudinal studies suggest a bidirectional relation between children’s psychopathology and PS (13, 14), and that due to our cross-sectional study design, we cannot draw causal conclusions. Nevertheless, a longitudinal study found that children’s externalizing problems decreases if PS does (77). Thus, interventions for parents to reduce PS may indeed be useful to reduce psychopathology in children and the risk for TTMD.

### 5.2 Relational schemas in parents with a mental illness and the relationship to psychopathological symptoms in children

Confirming our hypothesis, parents with a mental illness showed more negative and less positive RS than parents without a mental illness. This is in line with findings suggesting that parental psychopathological symptoms were positively associated with negative RS. Moreover, we found that parents with a mental illness showed less positive RS. A high extent of negative and a low extent of positive RS may discriminate between sub-clinical and clinically relevant psychopathological symptoms in parents, indicating this constellation as a potential risk factor for TTMD. Since means of self-reported PS in our sample were within the average range, differences in RS may not exclusively be due to heightened PS (39, 40). More likely, parents with a mental illness evaluated their children’s behavior in a more negative and less positive way due to their own psychopathology. This is in line with research suggesting that depressive symptoms in mothers lead to increased negative appraisals of their children (106), and a reduced threshold for tolerating aversive child behaviors (107). In line with our hypothesis, we found expected associations between RS and children’s psychopathological symptoms regardless of parents’ diagnostic status. This is consistent with previous findings on RS and psychopathological symptoms in children (49, 79, 80). In contrast to our study, the described studies examined clinically-referred child samples with externalizing behavior problems. We found that RS are associated with externalizing and internalizing symptoms as well although children’s psychopathology was within a normal range. In sum, our results support the idea that parental information processing can provide an explanation for how PS affects child outcomes. We need to mention again that the relation between children’s psychopathology and PS is bidirectional (13, 14), and that due to our cross-sectional study design, we cannot draw causal conclusions.

### 5.3 Psychophysiological arousal in parents with a mental illness and the relationship to psychopathological symptoms in children

To our knowledge, this is the first study examining psychophysiological arousal in parents with a mental illness. We did not differences between parents with and without a mental illness during baseline assessments or the FMSS in fundamental frequency range, rejecting our hypothesis. This could be due to several reasons. First, bivariate correlation analysis showed no significant associations between HR, HRV and fundamental frequency range. This is contrary to findings that fundamental frequency is a valid indicator of arousal correlating strongly with established indicators of ANS (63, 64). However, we found a significant difference between the baseline-period and FMSS, with parents showing higher fundamental frequency ranges during the baseline-period. Thus, arousal in parents differed between phases indicating a physiological stress response. Second, previous studies examined fundamental frequency during conflict tasks between couples or parents and their children. We assessed fundamental frequency during free speech in which parents talk freely about their child for five minutes, without the child being present. It could be that the arousal provoked in this task was not strong enough to elicit potential differences. We were not able to find any associations between fundamental frequency and children’s psychopathology either. This could be due to the described methodological reasons. Nevertheless, future studies should examine fundamental frequency in parents with a mental illness considering our limitations. Partially confirming our hypothesis, we found an interaction effect between group and phase in mean HR, indicating reduced reactivity in parents with a mental illness. As an indication of a physiological stress response, we found significant differences in mean HR between baseline-period and FMSS, with parents showing higher mean HR during FMSS. This is in line with findings on high-risk parents showing reduced reactivity in response to a stress task (56, 57). Contrary to previous studies, we were not able to find differences in HR between parents with and without a mental illness during baseline and FMSS. Comparing the mean HR during baseline with previous studies (56, 57), we need to mention that parents’ mean HR in our study was quite high (*M* = 80.71, *SD* = 9.58). We asked parents to describe a hidden object picture resulting in higher arousal compared to baseline obtained by asking parents to sit quietly. Speculatively, this makes it harder to detect any group differences. We did not detect reduced reactivity on HRV in parents with a mental illness, either. This could be due to further methodological reasons. First, we assessed HRV during FMSS to provoke a stress response, which is in contrast to studies using established stress tasks, such as a cry paradigm (57). In fact, we were not able to find significant changes in parents’ HRV in the baseline-period compared to FMSS. Probably, by using FMSS we did not induce enough stress to detect any changes in HRV. Unfortunately, we did not assess subjective stress response via questionnaire. Second, in contrast to previous studies, parents in our study were talking during the assessment of HRV. On the one hand, breathing and physical activity can affect HRV (108). On the other hand, we used RMSSD for calculating HRV because it is less affected by respiration (92). Third, we had a reduced sample size regarding HR and HRV. Since HRV is an important index for emotion regulation (53), we cannot conclude that emotion regulation is reduced in parents with a mental illness on a psychophysiological level. We were not able to detect significant associations between parents’ psychophysiological arousal and children’s psychopathological symptoms rejecting our hypothesis. This could be due to described methodological issues. Thus, the role of parents’ psychophysiological arousal in how PS affects child outcomes and in the TTMD remains unclear and should further be investigated.

## 6 Strength and limitations

The main strength of this study is the assessment of PS by using multimodal data. In this way, we used parents’ self-report, parents’ RS and psychophysiological measures to extend previous literature that is limited to studies using mainly parental self-report to assess PS (69). Another strength is the sample of parents with a mental illness with clinically verified diagnosis. Preventing reporter bias, our study can contribute to objectivity in research on PS and psychopathology in parents. Further, this was the first study examining the relation between PS in parents with a mental illness and psychopathological symptoms in children allowing the conclusion that PS seems to be relevant for TTMD. The large sample size and the representativeness of our clinical sample should also be mentioned. Aside from these strengths, several limitations warrant consideration when interpreting the findings from the present study. One limitation is that parents reported their children’s psychopathology. On the one hand, parents could overestimate their children’s psychopathological symptoms due to their own psychopathology (106). On the other hand, psychopathology of children was within the normal range. Moreover, parent-report has been shown to be more valid for externalizing symptoms than child-report (100, 109). Future research should consider this by using teacher-report for instance. Another limitation is the paradigm we used for the assessment of psychophysiological arousal. The FMSS is an established paradigm to assess RS, but it is not known as a stress paradigm to assess psychophysiological arousal. Future research should consider this by using an established stress paradigm, such as conflict tasks with parents and their children. This would allow assessing psychophysiological arousal in children as well. A final limitation of the study is that the data is cross-sectional and therefore does not allow causal conclusions. Therefore, longitudinal studies are needed to investigate how PS affects child outcomes. With respect to the role of PS in TTMD, longitudinal studies are needed as well to identify PS as a risk factor for TTMD. Given the longitudinal design of the COMPARE-study, this will be explored in a further study.

## 7 Conclusion and clinical implications

Our findings suggest that parents with a mental illness perceive increased PS, especially a lower extent of social support and less support in parental relationships, and that they evaluate their children in a more negative and less positive manner than parents without a mental illness. Regarding psychophysiological arousal, parents with a mental illness show a reduced reactivity in stress response, indicating a less flexible response of SNS. Beyond that, our findings suggest that perceived PS and the way, parents talk about their children are associated with children’s psychophysiological symptoms regardless of parental diagnostic status. Therefore, our results support the idea that parents and children benefit from specific (preventive) intervention programs to reduce PS (30). In this context, parenting programs have shown to be effective in reducing PS (110). In addition, our results support the idea, that these programs should consider how parents evaluate their children’s behavior. Besides, our results have several implications for clinical practice with parents with a mental illness. First, they indicate that therapeutic professionals should focus on interventions to increase social support (also in parental relationship) in parents with a mental illness. Such support should probably be differentiated between the amount of social support and perceived social support leading to different implications for interventions increasing social support. Furthermore, our result support the idea to facilitate access to support services, for example parenting programs. Second, the parent-child-relationship should be considered in therapeutic contexts. Since RS play a critical role in how parents read their children’s behavior, reframing caregiver’s beliefs about their children may be an important tool to modulate RS. Third, parents with a mental illness may profit from strategies to reduce stress and to increase coping skills, such as relaxation or biofeedback trainings. In sum, parents with a mental illness benefit from intervention programs to reduce PS and thus the risk for TTMD in their children.

## Data availability statement

The datasets presented in this article are not readily available because of patient confidentiality and restrictions by law. Requests to access the datasets should be directed to christina.schwenck@psychol.uni-giessen.de.

## Ethics statement

The studies involving humans were approved by ethics committee from Justus-Liebig-University Giessen, Philipps-University Marburg and Technical University Dortmund. The studies were conducted in accordance with the local legislation and institutional requirements. Written informed consent for participation in this study was provided by the participants’ legal guardians/next of kin.

## Author contributions

VS: Data curation, Formal analysis, Investigation, Writing – original draft, Validation. KH: Investigation, Writing – review & editing. RuS: Funding acquisition, Project administration, Writing – review & editing. SW: Funding acquisition, Methodology, Project administration, Writing – review & editing. HC: Funding acquisition, Methodology, Project administration, Writing – review & editing. MK: Funding acquisition, Project administration, Writing – review & editing. KO: Funding acquisition, Project administration, Writing – review & editing. CR: Funding acquisition, Project administration, Writing – review & editing. RiS: Funding acquisition, Project administration, Writing – review & editing. LW: Funding acquisition, Project administration, Writing – review & editing. AZ: Funding acquisition, Project administration, Writing – review & editing. CS: Funding acquisition, Methodology, Project administration, Supervision, Writing – review & editing.

## Group members of the COMPARE-Family Research Group

Study management: Stracke, Gilbert, Eitenmüller. Biometry and data management: Awounvo, Kirchner, Klose. Clinical study monitoring: Buntrock, Ebert. Recruiting center Bielefeld: Schlarb. Recruiting center Bochum: Margraf, Schneider, Friedrich, Teismann. Recruiting center Giessen: Stark, Metzger. Recruiting center Greifswald: Brakemeier, Wardenga, Hauck. Recruiting center Landau: Glombiewski, Schröder, Heider. Recruiting center Mainz: Jungmann, Witthöft. Recruiting center Marburg: Rief, Eitenmüller.
